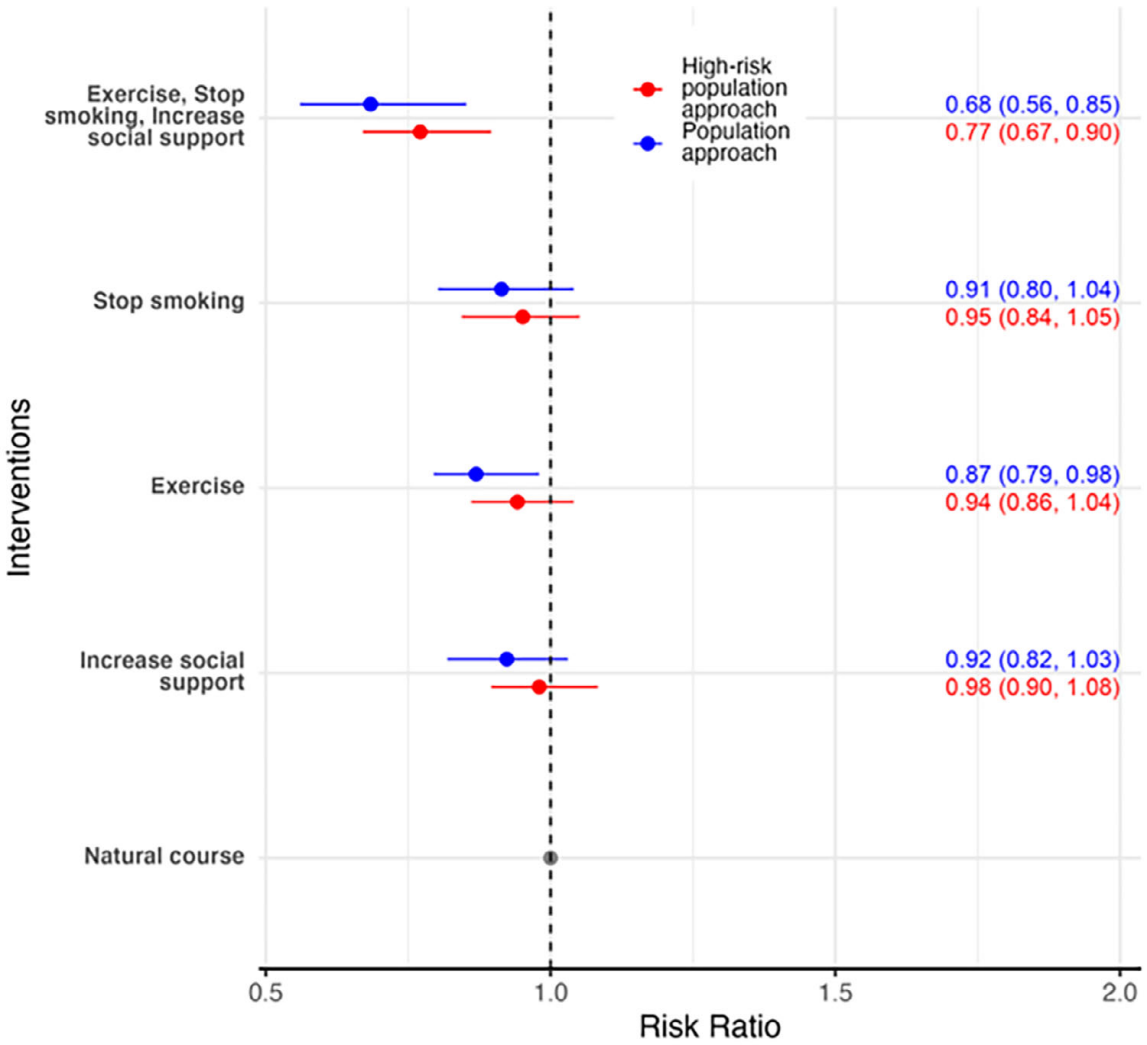# Hypothetical Midlife Lifestyle Interventions and Dementia Risk in US Older Adults: A Comparative Effectiveness Microsimulation Study

**DOI:** 10.1002/alz.090580

**Published:** 2025-01-09

**Authors:** Roch A Nianogo, Nicola A Churchill, Deborah E Barnes

**Affiliations:** ^1^ UCLA Fielding School of Public Health, University of California, Los Angeles, CA USA; ^2^ University of California, Los Angeles, Los Angeles, CA USA; ^3^ University of California, San Francisco, and San Francisco VA Health Care System, San Francisco, CA USA; ^4^ University of California, San Francisco, San Francisco, CA USA

## Abstract

**Background:**

Lifestyle and metabolic multi‐domain approaches targeting several risk factors at a time are increasingly being recognized as critical for dementia prevention since single‐factor approaches and one‐size‐fits‐all interventions often fall short in substantially reducing the dementia burden. Therefore, we compared the effectiveness of several hypothetical midlife lifestyle interventions considered singly and in combination across two health approaches: high‐risk subpopulations (targeted) and general population (untargeted).

**Method:**

Data came from the combined 2006 and 2008 biomarker samples of the Health and Retirement Study (HRS, N = 12,219). High‐risk populations (47%) were defined as individuals who: i) were APOEe4 carrier, ii) had high allostatic load (i.e., being in the top decile of the sum of z‐scores for biomarkers including C‐reactive Protein, HbA1c, systolic blood pressure), or iii) had metabolic syndrome (defined according to the Joint Interim Statement specification for metabolic syndrome based on measured criteria). We used the parametric, simulation‐based g‐formula to project the risk of dementia among older adults under different scenarios compared to the natural course of no‐intervention. We simulated three hypothetical lifestyle interventions based on being the top three lifestyle risk factors from the literature and the availability of the variables in HRS.

**Result:**

For single‐factor, untargeted interventions, only exercise was associated with significant reduction in dementia risk of 13% (95% CI: 2% to 21%) reduction. Increasing social support alone reduced the risk of dementia by 9% (95% CI: ‐4% to 20%) and smoking cessation alone by 8% (95% CI: ‐3% to 18%). When exercise was combined with smoking cessation and social support, there was a 32% (95% CI: 15% to 44%) reduction in dementia risk using the general population approach (untargeted) and a 23% (95% CI: 10% to 37%) reduction in dementia risk in the general population when targeting high‐risk individuals (targeted).

**Conclusion:**

Microsimulations suggest that midlife lifestyle interventions are likely to be marginally effective at reducing dementia risk when implemented individually with exercise being the most effective. Combined, multi‐domain approaches targeting several lifestyle factors at a time and targeting the general population regardless of risk factor distribution yielded a greater reduction in dementia risk than single lifestyle interventions or targeting only high‐risk individuals.